# Clinical and Genetic Analyses of 38 Chinese Patients with Peutz-Jeghers Syndrome

**DOI:** 10.1155/2020/9159315

**Published:** 2020-05-11

**Authors:** Bo-Da Wu, Yong-Jun Wang, Liang-Liang Fan, Hui Huang, Peng Zhou, Mei Yang, Xiao-Liu Shi

**Affiliations:** ^1^Department of Medical Genetics, The Second Xiangya Hospital, Central South University, Changsha, Hunan 410011, China; ^2^Department of Gastroenterology, The Second Xiangya Hospital, Central South University, Changsha, Hunan 410011, China; ^3^Department of Cell Biology, School of Life Science, Central South University, Changsha, Hunan 410012, China; ^4^Department of Pathology, The Second Xiangya Hospital, Central South University, Changsha, Hunan 410011, China

## Abstract

**Background:**

Peutz-Jeghers syndrome (PJS) is a rare autosomal dominant inherited disease caused by a germline mutation in the *STK11* gene. It is characterized by mucocutaneous pigmentation, gastrointestinal hamartomatous polyps, and cancer predisposition.

**Aims:**

We aimed to summarize the main clinical and genetic features of Chinese PJS patients and assessed the genotype-phenotype correlations.

**Methods:**

Thirty-eight patients clinically diagnosed with Peutz-Jeghers syndrome were included in this study from 2016 to 2019. Combined direct sequencing and multiplex ligation-dependent probe amplification tests were used to detect germline heterogeneous *STK11* mutations. RNA sequencing was performed in polyps of PJS patients and control groups to evaluate the difference in expression of *STK11*. The genotype-phenotype correlations were calculated by Kaplan-Meier analyses.

**Results:**

All 26 probands and 12 affected relatives had germline heterogeneous *STK11* mutations among which 8 variants were novel. Individuals with missense mutations had their first surgery and other symptoms significantly later than individuals with null mutations.

**Conclusion:**

This study expanded the spectrum of *STK11* gene mutations and further elucidated individuals with null mutations of *STK11* typically had an earlier onset of PJS symptoms and needed earlier management.

## 1. Introduction

Peutz-Jeghers syndrome (PJS, OMIM 175200) is a rare inherited autosomal dominant disease, with a triad of mucocutaneous pigmentation (MP), gastrointestinal hamartomatous polyps, and an increasing risk of a wide variety of malignancies [1–3]. The pathological type of PJS polyps is hamartomatous polyp, also known as Peutz-Jeghers-type hamartomatous polyp. Its histopathological features are peculiar branching-tree arrangement of the smooth muscle extending into the lamina propria. The incidence of this disease has been estimated to be 1 in 8,300 to 1 in 200,000 births [4].

The *STK11* (also named *LKB1*) gene mutation is responsible for PJS [5, 6]. It is located on 19p13.3 and comprises 9 coding exons and 1 noncoding exon, coding a member of the serine/threonine kinase family with 433 amino acids [6, 7]. *STK11* is a master tumor suppressor gene that regulates cellular responses involved in cell polarity, energy metabolism, and cell growth via different signaling pathways, including the LKB1/AMPK/mTOR pathway [8–10]. Approximately 80%-94% of PJS patients have germline STK11 mutations detected by direct sequencing and multiplex ligation-dependent probe amplification (MLPA) [11, 12].

The mucocutaneous pigmentation of PJS individuals only affects the appearance and does not require special treatment. The main hazards of PJS are polyp-associated complications, including abdominal pain, gastrointestinal bleeding (GIB), intestinal obstruction, and the occurrence of various malignancies. Patients often come to the hospital because of these symptoms and sometimes need emergency surgery. There are several cohort studies of the clinical and genetic characteristics of Chinese PJS patients. Jiang et al. reported that 34 of 47 PJS probands were tested *STK11* mutations using a combined strategy and found 25 mutations [13]. Wang et al. revealed that *STK11* Domain XI Mutations correlated with a very high incidence of dysplastic GI hamartomatous polyps [14], but the genotype-phenotype correlations are still not very clear.

This study aimed to summarize the clinical and genetic characteristics of Chinese PJS individuals, further explore the phenotype-genotype correlations of PJS and provide genetic counseling.

## 2. Materials and Methods

### 2.1. Patients and Sample Collection

A total of 38 PJS patients from 26 families who visited the Second Xiangya Hospital of Central South University from January 2016 to October 2019 were enrolled in this study and numbered 1-38. A clinical diagnosis of PJS was made when an individual had two or more of the following features: (1) two or more histologically confirmed Peutz-Jeghers-type hamartomatous polyps; (2) mucocutaneous hyperpigmentation of the mouth, lips, nose, eyes, genitalia, or fingers; and (3) family history of PJS [15]. All patients except one fulfilled the diagnostic criteria. This 7-year-old girl was suspected of having PJS because of typical PJS mucocutaneous pigmentation only, but she was too young to undergo any intestinal-associated testing. The collection of clinical data included demographics, history taking, family history, symptoms, PJS-related surgical interventions, and pathological data collection.

### 2.2. Genetic Testing

#### 2.2.1. Germline Mutation

Peripheral blood samples were collected from all 38 patients and their healthy relatives. Genomic DNA (gDNA) was prepared using the QIAamp DNA Blood Mini Kit (QIAGEN, Germany). We used the online website Primer 3 to design the primers (Supplementary Table 1). All 9 exons and flanking intronic sequences of *STK11* (RefSeq: NM_000455) were amplified with polymerase chain reaction (PCR), and all PCR products were sequenced directly using an ABI 3730XL Genetic Analyzer (ABI, Japan). Several online programs, including MutationTaster, SIFT, and PolyPhen-2, were used to predict the damaging effects of the variants. The interpretations of variants were assessed under the protocol issued by the ACMG guidelines [16]. If pathogenic/likely pathogenic variants were not detected in patients by direct sequencing, multiplex ligation-dependent probe amplification (MLPA) was performed to screen for exon deletions of the *STK11* gene. Techniques and methods for the MLPA test were provided by Kangso Medical Inspection, China.

Peripheral blood of a pregnant woman whose husband was a PJS patient with *STK11* c.527A>G pathogenic mutation was collected to do noninvasive prenatal testing (NIPT). The testing was carried out by BerryGenomics, China.

#### 2.2.2. RNA Sequencing

mRNA sequencing was performed in colonic polyps of 6 unrelated PJS patients, colonic mucosae of 6 non-PJS patients, and colonic polyps of 6 non-PJS patients. Techniques and methods for mRNA sequencing were carried out by OE Biotech (Shanghai, China). Briefly, mRNA was extracted using the mirVana miRNA Isolation Kit (Ambion, AM1561) following the manufacturer's protocol. An Agilent 2100 Bioanalyzer (Agilent Technologies, Santa Clara, CA, USA) was used to evaluate RNA integrity. The libraries were constructed using the TruSeq Stranded mRNA LTSample Prep Kit (Illumina, San Diego, CA, USA) according to the manufacturer's instructions. Then, these libraries were sequenced on the Illumina sequencing platform (HiSeq X Ten), and 150 bp paired-end reads were generated. Hisat2 (version 2.2.1.0) was used to align the reads to the genome and calculate the RPKM (reads per kilobase per million) values through Cufflinks (version 2.2.1).

### 2.3. Statistical Analysis

The software package SPSS Statistics Version 19.0 (IBM Corp., Armonk, New York, USA) was used for all of the analyses, and the data are presented as the mean ± SD. *P* values <0.05 were considered statistically significant. The difference in *STK11* expression was evaluated by *t* test. Differences in the time to onset of first reported gastrointestinal (GI) polyps, intestinal obstruction, and surgery between patients with missense mutations and those with null mutations were compared by Kaplan-Meier analysis, and a log-rank test was used.

## 3. Results

### 3.1. Clinical Features

Thirty-eight PJS patients from 26 unrelated families were included in the study, consisting of 15 familial and 11 sporadic PJS cases (Supplementary Figure 1). There were 22 (65%) male and 14 (35%) female patients, aged from 2 to 57 years (27.2 ± 13.2) (Table 1).

**Figure 1:**
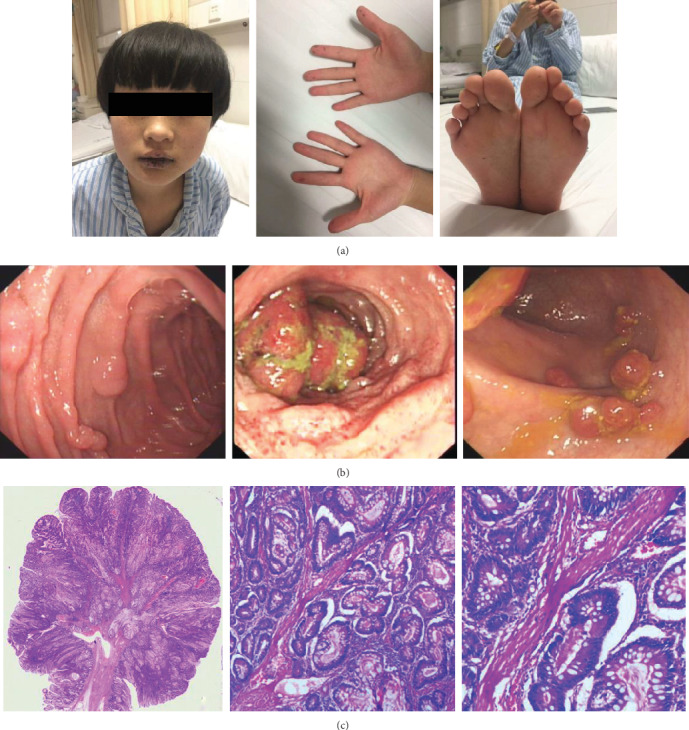
The main clinical characteristics of PJS patients. (a) Mucocutaneous pigmentation (patient 24). (b) Gastrointestinal endoscopy images showed polyps in the stomach, ileum, and colon (patient 8). (c) Histopathology presented Peutz-Jeghers-type hamartomatous polyp with peculiar branching-tree arrangement of the smooth muscle extending into the lamina propria (patient 7).

All 38 patients had mucocutaneous pigmentation before 7 years old, which mostly occurred around the lip, buccal mucosa, fingers, and toes (Figure 1(a)). Most of them did not perform any intervention, and some had laser cosmetic surgeries. Some patients reported a tendency for oral pigmentation to fade when they were in their 30s. Only 3 patients (8%) were considered with PJS because of mucocutaneous pigmentations, and the rest were diagnosed with PJS for gastrointestinal symptoms (66%) or PJS family history (26%) (Table 1).

Twenty-six patients (68%) had a history of abdominal pain, 18 patients (47%) had hematochezia, and most of them had occult blood loss. Thirty-two patients (84%) had undergone procedures to detect gastrointestinal polyps (the other 6 patients did not receive any GI investigations because they were too young or had no symptoms) (Table 1). These polyps were sessile or pedunculated, and their sizes ranged from a few millimeters to several centimeters (Figure 1(b)). The largest one was 7.0 cm, and most polyps were 0.2-2.0 cm. The mean age of the first detection of gastrointestinal polyps was 18 years old (2-43 years old), with intestinal obstruction as the most frequent reason for detection. Seventeen patients (45%) had a history of one or more episodes of intestinal obstruction due to GI polyps, and the mean age of the first occurrence of intestinal obstruction was 18 years old (11-31 years). Nineteen PJS patients (50%) had undergone at least one PJS-related surgery at a mean age at the first surgery of 17.5 years old (5-26 years). Twenty-four out of 29 operations (83%) were performed because of intestinal obstruction. Detailed information is shown in Supplementary Table 2.

Three malignant tumors were discovered in surveillance in 3 different PJS patients (one sinus cancer (T1N0M0), one breast cancer (Tis), and one colon cancer (T2N0M0)), and all of them received surgical resection.

We obtained a total of 52 gastrointestinal polyp tissues from the pathology archives of the Second Xiangya Hospital. These polyps were resected endoscopically or surgically from 21 PJS patients, and the pathological diagnosis was made by trained pathologists through reevaluation of the pathology slides. Twenty-five polyps from 19 patients were consistent with the histologic features of Peutz-Jeghers-type hamartomatous polyps, characterized by cores of smooth muscle fibers in a tree-like pattern with hyperplasia of the epithelium (Figure 1(c)). Other pathological types of GI polyps, including adenomatous, tubule-villous, hyperplastic, inflammatory, and mixed polyps, were also detected in PJS individuals.

### 3.2. Genetic Analyses

**Figure 2:**
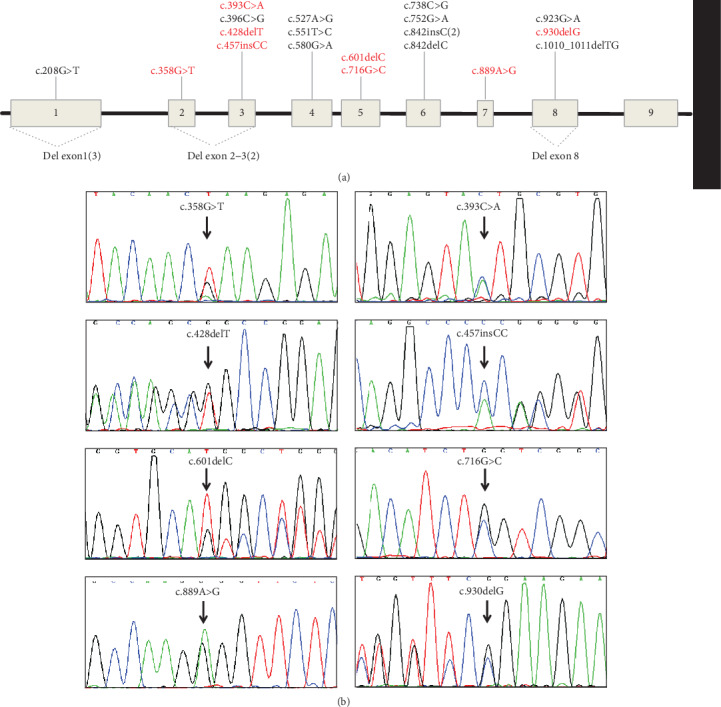
Variant spectrum of the *STK11* gene detected in our study. (a) Schematic of the localization of 22 *STK11* mutations; the numbers in parentheses indicate that the variation existed in several unrelated families. (b) Chromatogram of 8 novel *STK11* gene mutations; mutations are indicated by arrows.

Germline mutation screening of the *STK11* gene was performed in 26 probands (15 familial probands and 11 sporadic probands). A pathogenic or likely pathogenic variant was detected in all probands with a mutation detection rate of 100%. Twenty variants were nucleotide substitutions or indels that were detected by Sanger sequencing (7 were missense variants, and 13 variants were truncating) (Figure 2, Supplementary Figure 2). All mutations fell within the coding region spanning exon 1 and exon 8, and no mutation in exon 9 was identified in any of these PJS individuals (Figure 2(a)). These variants were not present in the 1000 Genomes Project database (http://browser.1000genomes.org) or the Exome Aggregation Consortium database (http://exac.broadinstitute.org/). Several online programs, including MutationTaster, SIFT, and polyphen2, predicted that these variants had deleterious effects on the gene product. All of these variants occurred within a highly conserved amino acid. According to the ACMG guidelines, these mutations were classified as pathogenic or likely pathogenic. Among these variants, one (c.841_842insC) recurred in 2 unrelated probands, and 18 variants were unique. Eight variants (c.393C>A, c.358G>T, c.428_428delT, c.457_458insCC, c.601_601delC, c.716G>C, c.889A>G, c.930delG) have not yet been reported (Figure 2(b)).

**Figure 3:**
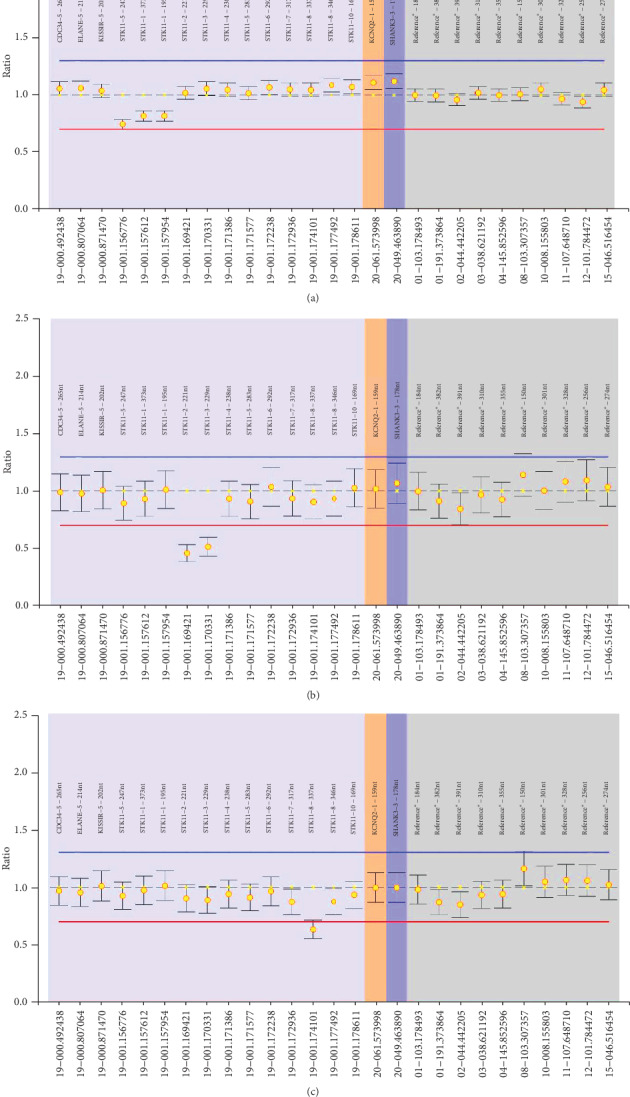
Large *STK11* gene mutations detected by multiplex ligation-dependent probe amplification in patients with PJS. (a–c) The deletion of exon 1, exons 2-3, and exon 8, respectively.

The remaining 6 variants were large partial gene deletions detected by MLPA. Three deletions encompassed exon 1, 2 deletions spanned exons 2-3, and the other deletion encompassed exon 8 (Figure 3).

Germline mutation testing of *STK11* was extended to the probands' relatives. Thus, when affected relatives were included, a total of 38 PJS patients had a *STK11* pathogenic/likely pathogenic variant in our study (15 familial probands, 11 sporadic cases, and 12 relatives) (Supplementary Table 2).

Regrettably, the NIPT demonstrated the fetus had a same heterozygous variation of *STK11* c.527A>G as the father which indicated that the baby is likely to be a PJS patient. But the couple still decided to give birth to the child with closely follow up on PJS symptoms after birth. The Sanger sequencing of *STK11* gene using the baby's peripheral blood after birth further confirmed that he did have this mutation, and mucocutaneous pigmentation occurred around the lip at the age of 1 year.

**Figure 4:**
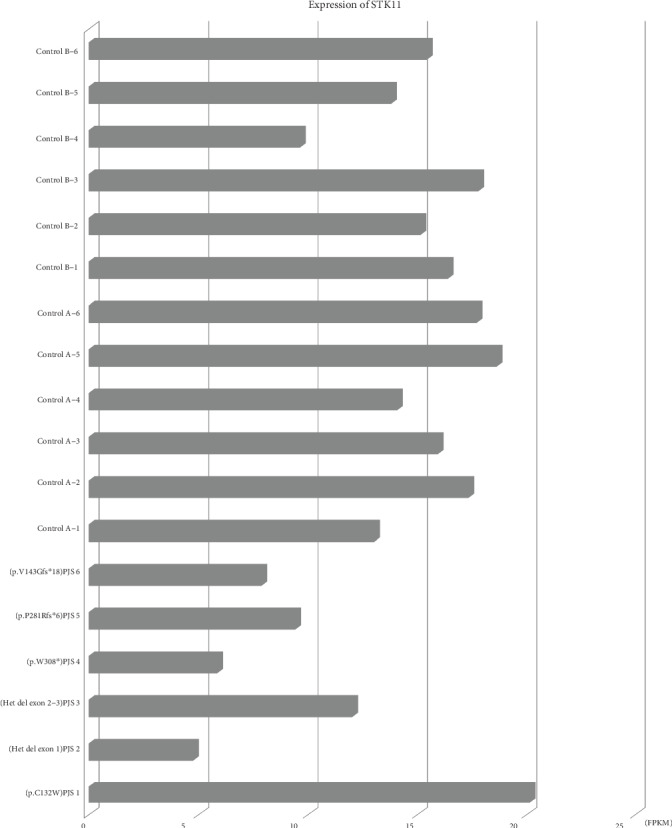
The expression of *STK11*. The abscissa indicates the expression level of the gene, which is represented by FPKM. The ordinate represents different samples. The difference in *STK11* expression was evaluated by *t* test. The *STK11* gene expression in colonic polyps of patients with a null *STK11* mutation (mean ± SD: 8.0 ± 2.9) was significantly decreased compared with control A (mean ± SD: 16.1 ± 2.2, *P* = 0.0005) and control B (mean ± SD: 14.7 ± 2.8, *P* = 0.004).

RNA sequencing was performed on colonic polyps of 6 unrelated PJS patients (including 1 missense mutation and 5 null mutations) and colonic mucosae of 6 non-PJS patients (control A group) and colonic polyps of 6 non-PJS patients (control B group) (Figure 4). The difference in *STK11* gene expression was analyzed between these 5 null mutations and control groups A and B. The *STK11* gene expression in colonic polyps of patients with null *STK11* mutation (8.0 ± 2.9) was significantly decreased compared with control A (16.1 ± 2.2, *P* = 0.0005) and control B (14.7 ± 2.8, *P* = 0.004).

### 3.3. Genotype/Phenotype Correlations

PJS patients (including index patients and relatives) were subdivided into two groups to explore genotype-phenotype correlations: individuals with missense mutations (MM group, *n* = 15, mean age 29.8 ± 14.7) and individuals with null mutations, including truncating and large deletion variants (NM group, *n* = 23, mean age 25.3 ± 12.4).

**Figure 5:**
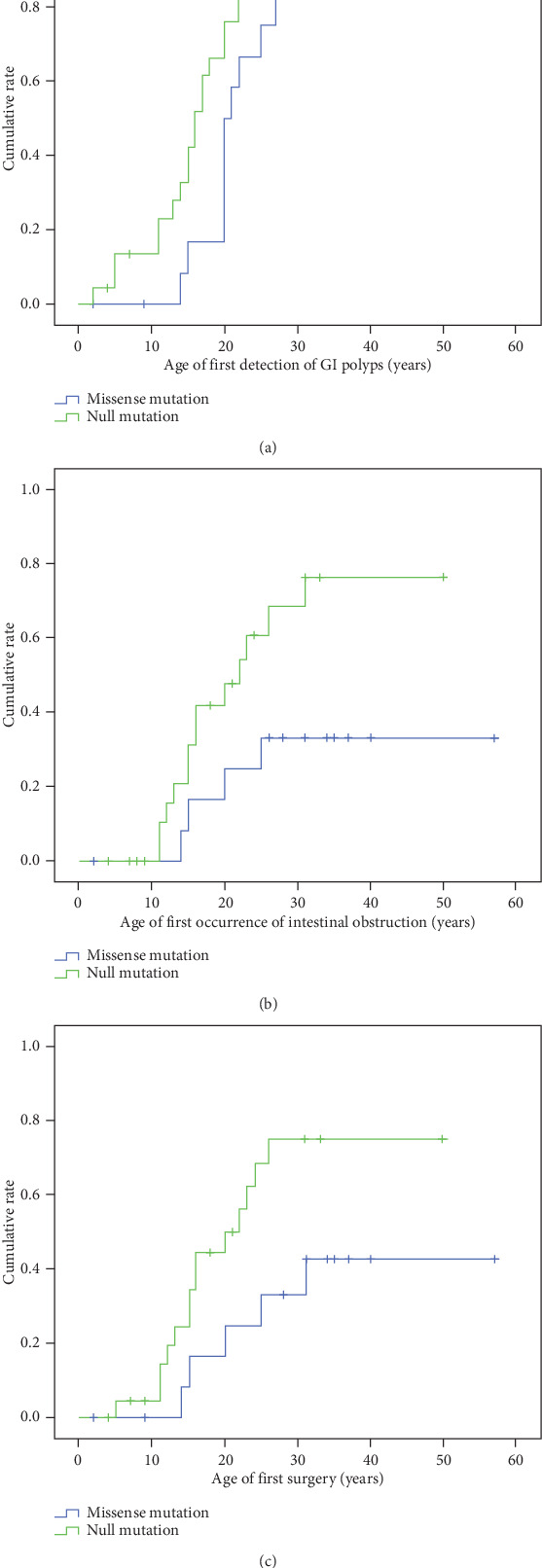
Differences in the time to onset of first reported gastrointestinal symptoms or related surgery between patients with missense mutations and those with null mutations were compared by Kaplan-Meier analysis and the log-rank test. (a) The age of first detection of GI polyps (mean ± SD of missense group vs null group: 21.2 ± 4.5 vs 17.7 ± 8.6, *P* = 0.04). (b) The age of first occurrence of intestinal obstruction (mean ± SD18.5 ± 5.1 vs 17.8 ± 6.2, *P* = 0.036). (c) The age of first surgery (mean ± SD21.00 ± 7.106 vs 16.36 ± 5.956, *P* = 0.049).

In the MM group, 4 patients (27%) including a 57-year-old man did not have any GI symptoms and did not receive any GI test. In the NM group, only 2 children (9%) did not undergo any test to detect GI polyps. We found that the time of the first detection of gastrointestinal polyps was significantly earlier in the NM group than the MM group (missense vs null group: 21.2±4.5 vs 17.7±8.6, *P*=0.04) (Figure 5(a)).

Four patients had a history of intestinal obstruction in the MM group (27%), and 13 patients had a history of intestinal obstruction in the NM group (57%). There was a significant difference in the age of first-onset intestinal obstruction between patients with missense mutations and those with null mutations (18.5 ± 5.1 vs 17.8 ± 6.2*P* = 0.036) (Figure 5(b)).

Five patients with missense mutations (33%) and 14 patients with null mutations had undergone at least one surgery (61%). Comparing the age of the first surgery in the two patient groups, individuals with missense mutations had their first surgery significantly later than patients with null mutations (21.00 ± 7.106 vs 16.36 ± 5.956, *P* = 0.049) (Figure 5(c)).

## 4. Discussion

In this study, we enrolled 38 Chinese PJS patients, which included index cases and family members. All patients had germline heterogeneous *STK11* mutations, and 7 variants were novel. We summarized the main clinical and genetic features of these patients and discovered that individuals with missense mutations had their first surgery and other symptoms significantly later than null mutations. A fetus was identified as having the same heterozygous nonsense mutation in *STK11* as the father by noninvasive prenatal testing.

PJS is characterized by mucocutaneous pigmentation, hamartomatous polyposis, and an increasing risk of developing cancer. In our study, all patients had mucocutaneous pigmentation, and most of them did not undergo any intervention, and a few had laser cosmetic surgery. In PJS patients, polyps are found throughout the GI tract, which can cause significant complications, including abdominal pain, gastrointestinal bleeding, anemia, and especially intestinal obstruction. In 2011, Van Lier et al. studied 110 PJS patients, and they reported that 69% of PJS patients had at least one intussusception, and the mean age of the first occurrence was 16 years old. The intussusception risk was 50% at the age of 20 years, and 92.5% required surgery [17]. Hinds et al. reported that 68% of patients underwent laparotomy before the age of 18 because of intestinal obstruction [18]. This is consistent with our results. In our study, 17 of 38 PJS patients (45%) had a history of at least one episode of intestinal obstruction, and there were 26 events, and 24 (96%) required emergency laparotomy. The mean age of the first occurrence of intestinal obstruction was 18 years old (11-31 years). Ten out of these 17 patients (59%) experienced the first intestinal obstruction before 18 years old. It reminded us that PJS patients should undergo early gastrointestinal polyp screening to reduce complications and surgery events.

The other main clinical characteristic of PJS patients is the increasing risk of various carcinomas [19]. It was reported that the risk for developing cancer at ages 20, 30, 40, 50, 60, and 70 years was 2%, 5%, 17%, 31%, 60%, and 85%, respectively [20]. In our study, 3 PJS patients had malignant tumors. A 30-year-old PJS woman developed estrogen receptor (ER)-positive and Her2-positive breast cancer. A study of the Indian cohort of PJS showed that all 4 breast cancer cases with the *STK11* pathogenic variant were ER-positive and Her2-negative [21]. In the future, we will pay more attention to the development of tumors in PJS patients.

PJS is a rare inherited autosomal dominant disorder that is caused by *STK11* gene mutations. Combining the direct sequencing and MLPA test, we found that the germline *STK11* mutation rate of PJS patients in our study was 100%, which was higher than the 80–94% frequency reported in most previous studies. However, in 2010, Janos and his colleagues also reported a 100% detection rate of PJS [22]. The reason for this difference is unclear. It may be related to the different patient enrollment conditions. For example, in 2006, Volikos et al. reported that the detection rate of germline mutations in PJS patients was approximately 80%. The diagnosis of PJS in their study was made when the patient presented with two or more PJS polyps, one polyp and typical pigmented lesions, or one polyp and a family history of PJS [11]. Apparently, our diagnostic criteria were more rigorous.

*STK11* encodes an evolutionarily conserved serine/threonine kinase that includes 3 major domains: the N-terminal noncatalytic domain (1-49 aa), a catalytic kinase region (49-309 aa) and a C-terminal noncatalytic regulatory domain (309-433 aa). There are some autophosphorylation and phosphorylation sites in the 2 noncatalytic domains [23]. It is an important kinase involved in crucial cellular processes, such as cell growth, cell polarity, and energy metabolism. Loss-of-functions of LKB1 leads to PJS phenotypes. In our study, all missense mutations were detected in the serine/threonine kinase domain, which may result in impaired kinase activity and cell growth suppressive capacity [6, 24, 25].

Truncating mutations were expected to result in a loss of normal protein function through either protein truncation or nonsense-mediated mRNA decay (NMD). Our study showed that null mutations had lower *STK11* expression, while missense mutations did not influence the expression of *STK11*. Therefore, null mutations may cause greater damage to the gene product and may cause a more severe phenotype.

However, genotype-phenotype studies of PJS individuals are contradictory. Van Lier and his colleagues discovered that the incidence of intussusception is not influenced by *STK11* mutation status [17]. Amos et al. found that individuals with missense mutations had significantly later onset of the first polypectomy and of other symptoms when compared with those participants with either truncating mutations or no detectable mutation [26]. Salloch et al. discovered that patients with truncation mutations require more surgical GI interventions and tend to develop more polyps and cancers [27]. Our study similarly indicated that individuals with null mutations of *STK11* had earlier onset for PJS symptoms, including intestinal obstruction and first operation events, than those with missense mutations. This revealed that patients with null variations needed earlier management to prevent complications such as intestinal obstruction, reducing the laparotomy events.

The purpose of gene testing is to promote genetic diagnosis and counseling of inherited diseases. For individuals with suspected PJS or relatives of confirmed PJS patients, it is important to detect *STK11* gene mutations to make early diagnoses and treatments to reduce the occurrence of GI complications and malignancies. In addition, as PJS is an autosomal dominant inherited disease, there is a 50% risk of inheriting the *STK11* variant for the children of an individual with a *STK11* pathogenic/likely pathogenic mutation. It is possible to make a diagnosis as early as pregnancy or even prepregnancy through prenatal testing or preimplantation genetic diagnosis. In our study, we identified a fetus harboring the pathogenic variant of *STK11* through NIPT, but the parents chose to give birth to this fetus and closely monitor after birth. *STK11* gene testing of peripheral blood after birth confirmed that he inherited the pathogenic mutation from his father. Now the child is 2 years old, and the melanin spots appeared on the lips approximately 1 year old and gradually increased. The other two PJS families in our study are trying to perform preimplantation genetic diagnosis. There are broad application prospects for prenatal testing and preimplantation genetic diagnosis.

In summary, our study deepened the understanding of the clinical and genetic characteristics of PJS patients in China, expanded the spectrum of *STK11* gene mutations, and further elucidated the necessity of *STK11* testing of individuals with suspected PJS or at-risk relatives. Typically, individuals with null mutations of *STK11* had earlier onset of PJS symptoms and needed early intervention.

## Figures and Tables

**Table 1:** Clinical characteristics of 38 PJS patients.

Characteristic	Patients (*n* = 38)
Demographics, *n* (%)	
Familial probands	15 (39%)
Relatives	12 (32%)
Sporadic	11 (29%)
Female	15 (40%)
Male	23 (60%)
Symptoms, *n* (%)	
Mucocutaneous pigmentation	38 (100%)
Gastrointestinal polyps	32 (84%)
Abdominal pain	26 (68%)
Surgery	19 (50%)
Gastrointestinal bleeding	18 (47%)
Intestinal construction	17 (45%)
Malignancy	3 (8%)
Reason of initial evaluation, *n* (%)	
Intestinal construction	14 (37%)
Family history	10 (26%)
Gastrointestinal bleeding	9 (24%)
Mucocutaneous pigmentation	3 (8%)
Diarrhea	2 (5%)

## Data Availability

The data supporting the conclusions are included in the article. Raw data are available upon request.

## Supplementary Materials

Supplementary Materials Supplementary Figure 1: The pedigree of 26 families. Supplementary Figure 2: Chromatogram of 11 other *STK11* gene mutations. Supplementary Table 1: Primers of the *STK11* gene. Supplementary Table 2: Detailed clinical and genetic data for patients with PJS.

## References

[1] Bruwer A., Bargen J. A., Kierland R. R. (1954). Surface pigmentation and generalized intestinal polyposis; (Peutz-Jeghers syndrome). *Proceedings of the Staff Meetings Mayo Clinic*.

[2] Beggs A. D., Latchford A. R., Vasen H. F. A. (2010). Peutz-Jeghers syndrome: a systematic review and recommendations for management. *Gut*.

[3] Jeghers H., McKusick V. A., Katz K. H. (1949). Generalized intestinal polyposis and melanin spots of the oral mucosa, lips and digits; a syndrome of diagnostic significance. *The New England Journal of Medicine*.

[4] Giardiello F. M., Trimbath J. D. (2006). Peutz-Jeghers syndrome and management recommendations. *Clinical Gastroenterology and Hepatology*.

[5] Hemminki A., Markie D., Tomlinson I. (1998). A serine/threonine kinase gene defective in Peutz-Jeghers syndrome. *Nature*.

[6] Jenne D. E., Reomann H., Nezu J. I. (1998). Peutz-Jeghers syndrome is caused by mutations in a novel serine threoninekinase. *Nature Genetics*.

[7] Hemminki A., Tomlinson I., Markie D. (1997). Localization of a susceptibility locus for Peutz-Jeghers syndrome to 19p using comparative genomic hybridization and targeted linkage analysis. *Nature Genetics*.

[8] Zhao R. X., Xu Z. X. (2014). Targeting the LKB1 tumor suppressor. *Current Drug Targets*.

[9] Alessi D. R., Sakamoto K., Bayascas J. R. (2006). LKB1-dependent signaling pathways. *Annual Review of Biochemistry*.

[10] Ollila S., Domènech-Moreno E., Laajanen K. (2018). Stromal Lkb1 deficiency leads to gastrointestinal tumorigenesis involving the IL-11-JAK/STAT3 pathway. *The Journal of Clinical Investigation*.

[11] Volikos E., Robinson J., Aittomäki K. (2006). LKB1 exonic and whole gene deletions are a common cause of Peutz-Jeghers syndrome. *Journal of Medical Genetics*.

[12] Aretz S., Stienen D., Uhlhaas S. (2005). High proportion of large genomic STK11 deletions in Peutz-Jeghers syndrome. *Human Mutation*.

[13] Jiang Y. L., Zhao Z. Y., Li B. R., Wang H., Yu E. D., Ning S. B. (2019). STK11 gene analysis reveals a significant number of splice mutations in Chinese PJS patients. *Cancer Genetics*.

[14] Wang Z., Wu B., Mosig R. A. (2014). STK11 domain XI mutations: candidate genetic drivers leading to the development of dysplastic polyps in Peutz-Jeghers syndrome. *Human Mutation*.

[15] Provenzale D., Gupta S., Ahnen D. J. (2016). Genetic/familial high-risk assessment: colorectal version 1.2016, NCCN clinical practice guidelines in oncology. *Journal of the National Comprehensive Cancer Network*.

[16] Li M. M., Datto M., Duncavage E. J. (2017). Standards and guidelines for the interpretation and reporting of sequence variants in Cancer: a joint consensus recommendation of the Association for Molecular Pathology, American Society of Clinical Oncology, and College of American Pathologists. *The Journal of Molecular Diagnostics*.

[17] van Lier M. G. F., Mathus-Vliegen E. M. H., Wagner A., van Leerdam M. E., Kuipers E. J. (2011). High cumulative risk of intussusception in patients with Peutz-Jeghers syndrome: time to update surveillance guidelines?. *The American Journal of Gastroenterology*.

[18] Hinds R., Philp C., Hyer W., Fell J. M. (2004). Complications of childhood Peutz-Jeghers syndrome: implications for pediatric screening. *Journal of Pediatric Gastroenterology and Nutrition*.

[19] Resta N., Pierannunzio D., Lenato G. M. (2013). Cancer risk associated with STK11/LKB1 germline mutations in Peutz–Jeghers syndrome patients: Results of an Italian multicenter study. *Digestive and Liver Disease*.

[20] Hearle N., Schumacher V., Menko F. H. (2006). Frequency and spectrum of cancers in the Peutz-Jeghers syndrome. *Clinical Cancer Research*.

[21] Lipsa A., Kowtal P., Sarin R. (2019). Novel germline STK11 variants and breast cancer phenotype identified in an Indian cohort of Peutz-Jeghers syndrome. *Human Molecular Genetics*.

[22] Papp J., Kovacs M. E., Solyom S., Kasler M., Børresen-Dale A. L., Olah E. (2010). High prevalence of germline STK11mutations in Hungarian Peutz-Jeghers syndrome patients. *BMC Medical Genetics*.

[23] Sapkota G. P., Boudeau J., Deak M., Kieloch A., Morrice N., Alessi D. R. (2002). Identification and characterization of four novel phosphorylation sites (Ser31, Ser325, Thr336 and Thr366) on LKB1/STK11, the protein kinase mutated in Peutz-Jeghers cancer syndrome. *The Biochemical Journal*.

[24] Korsse S. E., Peppelenbosch M. P., van Veelen W. (2013). Targeting LKB1 signaling in cancer. *Biochimica et Biophysica Acta (BBA) - Reviews on Cancer*.

[25] Mehenni H., Gehrig C., Nezu J. I. (1998). Loss of LKB1 kinase activity in Peutz-Jeghers syndrome, and evidence for allelic and locus heterogeneity. *American Journal of Human Genetics*.

[26] Amos C. I., Keitheri-Cheteri M. B., Sabripour M. (2004). Genotype-phenotype correlations in Peutz-Jeghers syndrome. *Journal of Medical Genetics*.

[27] Salloch H., Reinacher-Schick A., Schulmann K. (2010). Truncating mutations in Peutz-Jeghers syndrome are associated with more polyps, surgical interventions and cancers. *International Journal of Colorectal Disease*.

