# Supplementary material for: Clinical and Genetic Analyses of 38 Chinese Patients with Peutz-Jeghers Syndrome
Source: Biomed Res Int. 2020 May 11;2020:9159315. doi: 10.1155/2020/9159315 (PMC7240661; doi:10.1155/2020/9159315)
Supplement: Supplementary Materials — Supplementary Figure 1: The pedigree of 26 families. Supplementary Figure 2: Chromatogram of 11 other STK11 gene mutations. Supplementary Table 1: Primers of the STK11 gene. Supplementary Table 2: Detailed clinical and genetic data for patients with PJS. [file 9159315.f1.zip › Figure 1 2 3 4 5+Supplementary Figure 1_BMRI_3076268.pptx]

## Slide 1
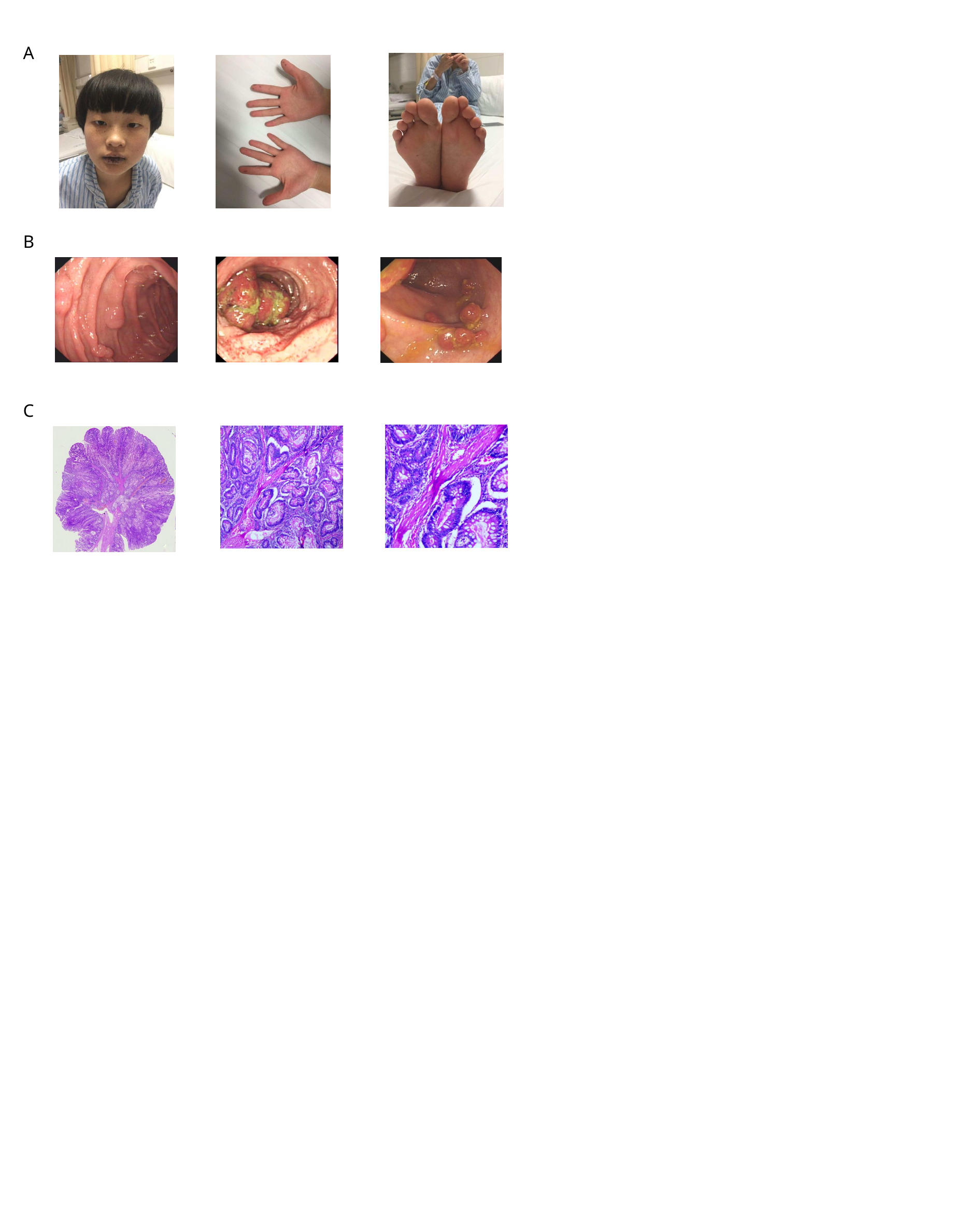

A
B
C

## Slide 2
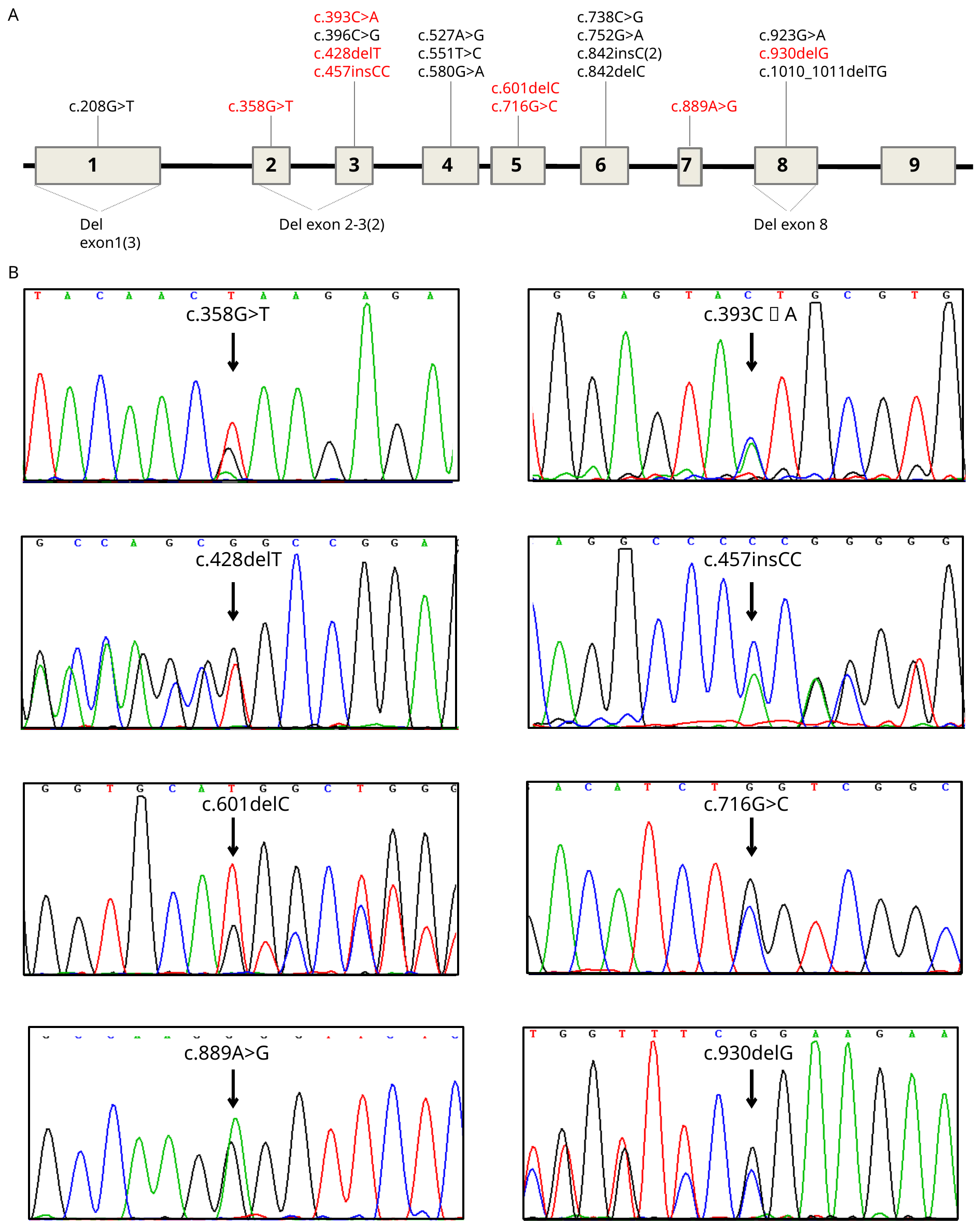

A
c.393C>A
c.396C>G
c.428delT
c.457insCC
c.738C>G
c.752G>A
c.842insC(2)
c.842delC
c.527A>G
c.551T>C
c.580G>A
c.923G>A
c.930delG
c.1010_1011delTG
c.601delC
c.716G>C
c.208G>T
c.358G>T
c.889A>G
1
 2
 3
 4
 5
 6
 7
 8
 9
Del exon1(3)
Del exon 2-3(2)
Del exon 8
B
c.358G>T
c.393C＞A
c.428delT
c.457insCC
c.601delC
c.716G>C
c.889A>G
c.930delG

## Slide 3
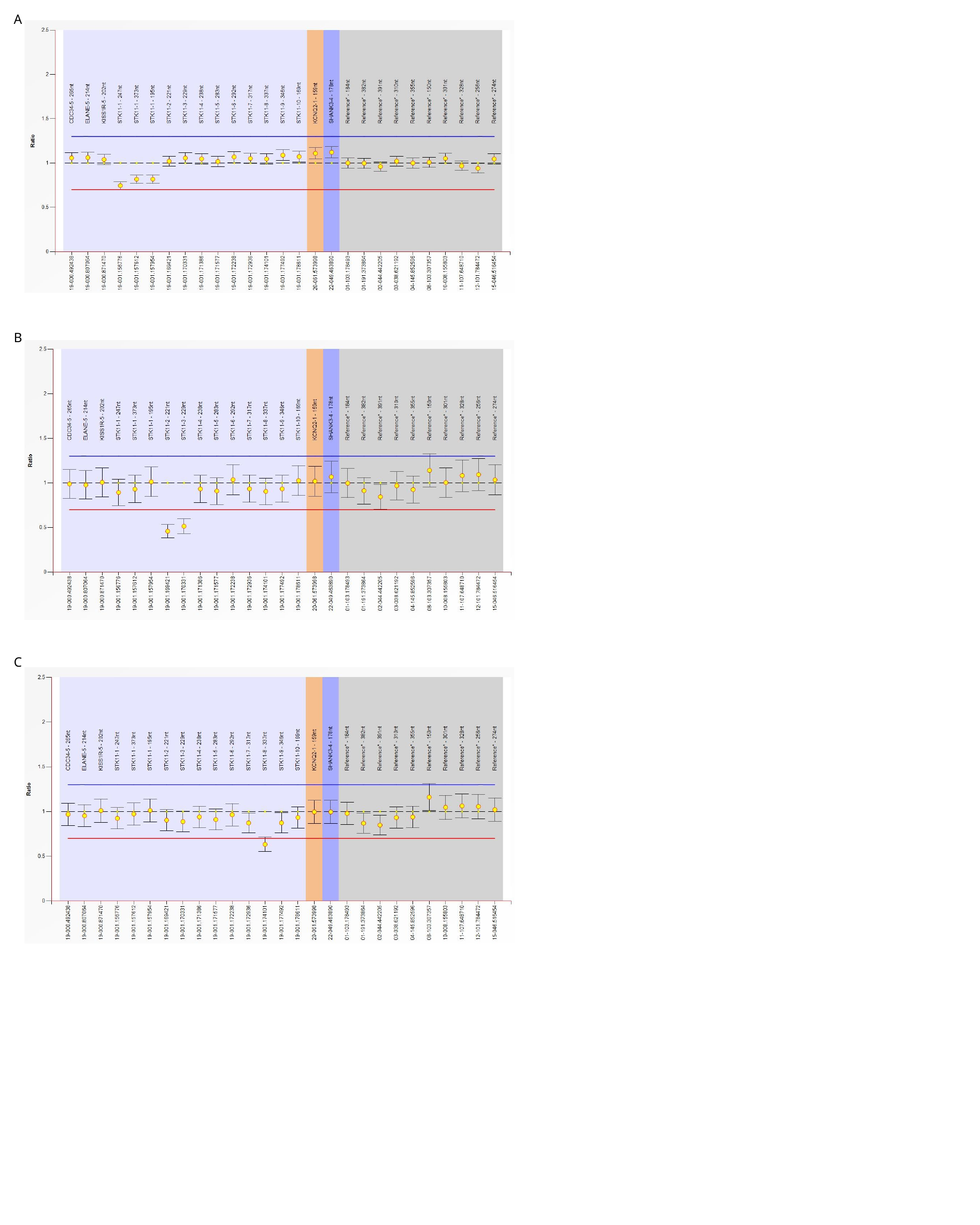

A
B
C

## Slide 4
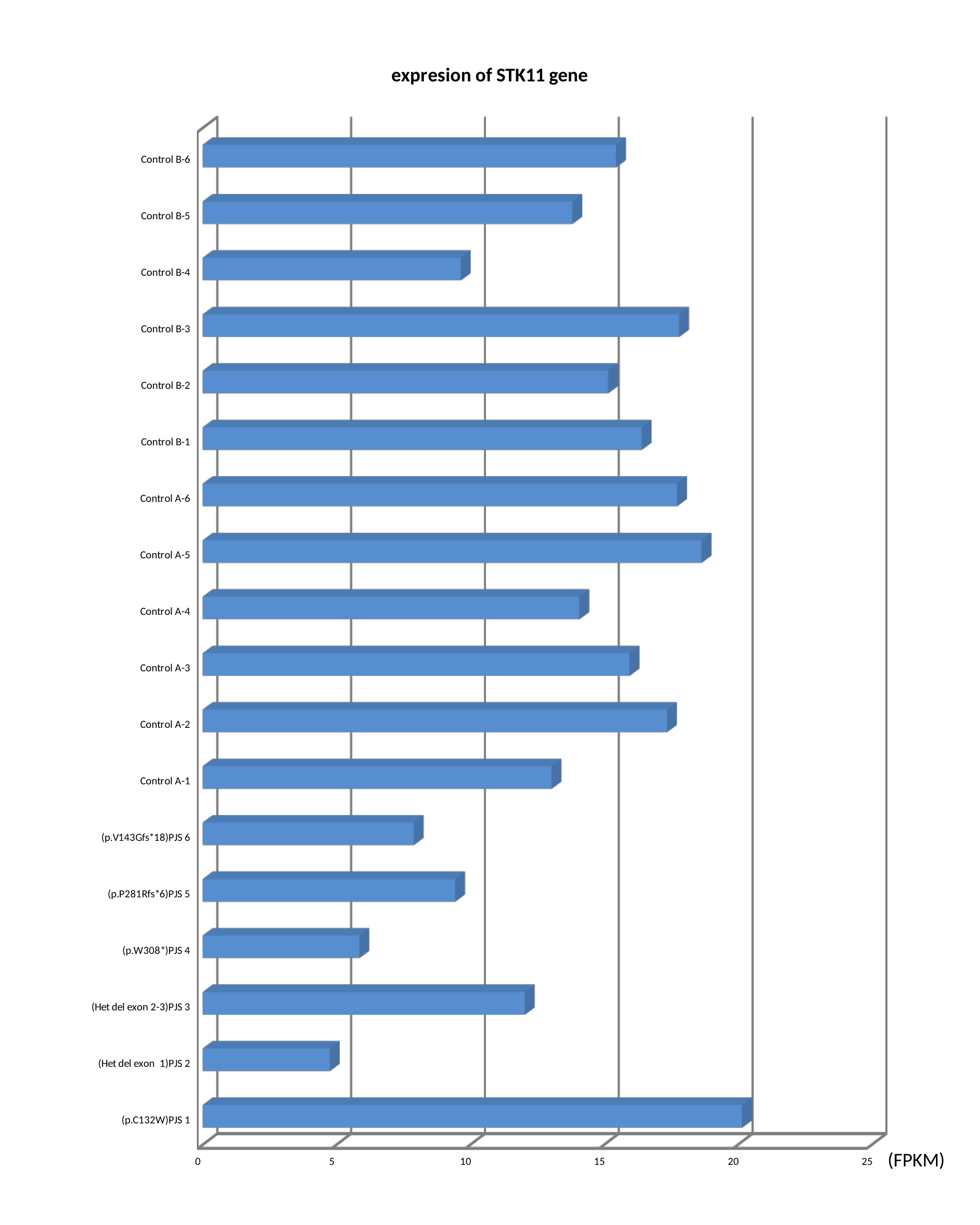

[unsupported chart]
(FPKM)

## Slide 5
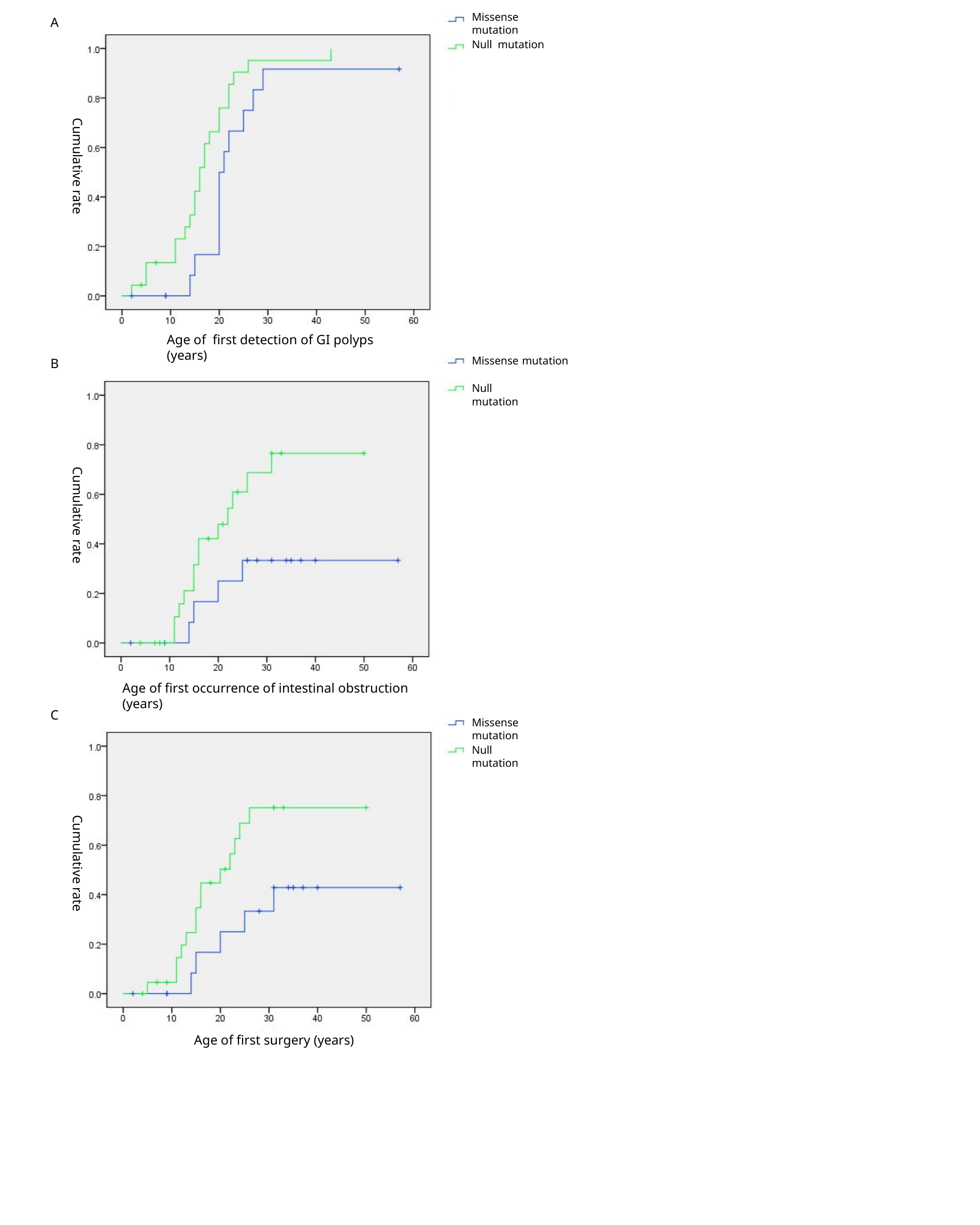

Missense mutation
Null mutation
A
Cumulative rate
Age of first detection of GI polyps (years)
Missense mutation
Null mutation
B
Cumulative rate
Age of first occurrence of intestinal obstruction (years)
C
Missense mutation
Null mutation
Cumulative rate
Age of first surgery (years)

## Slide 6
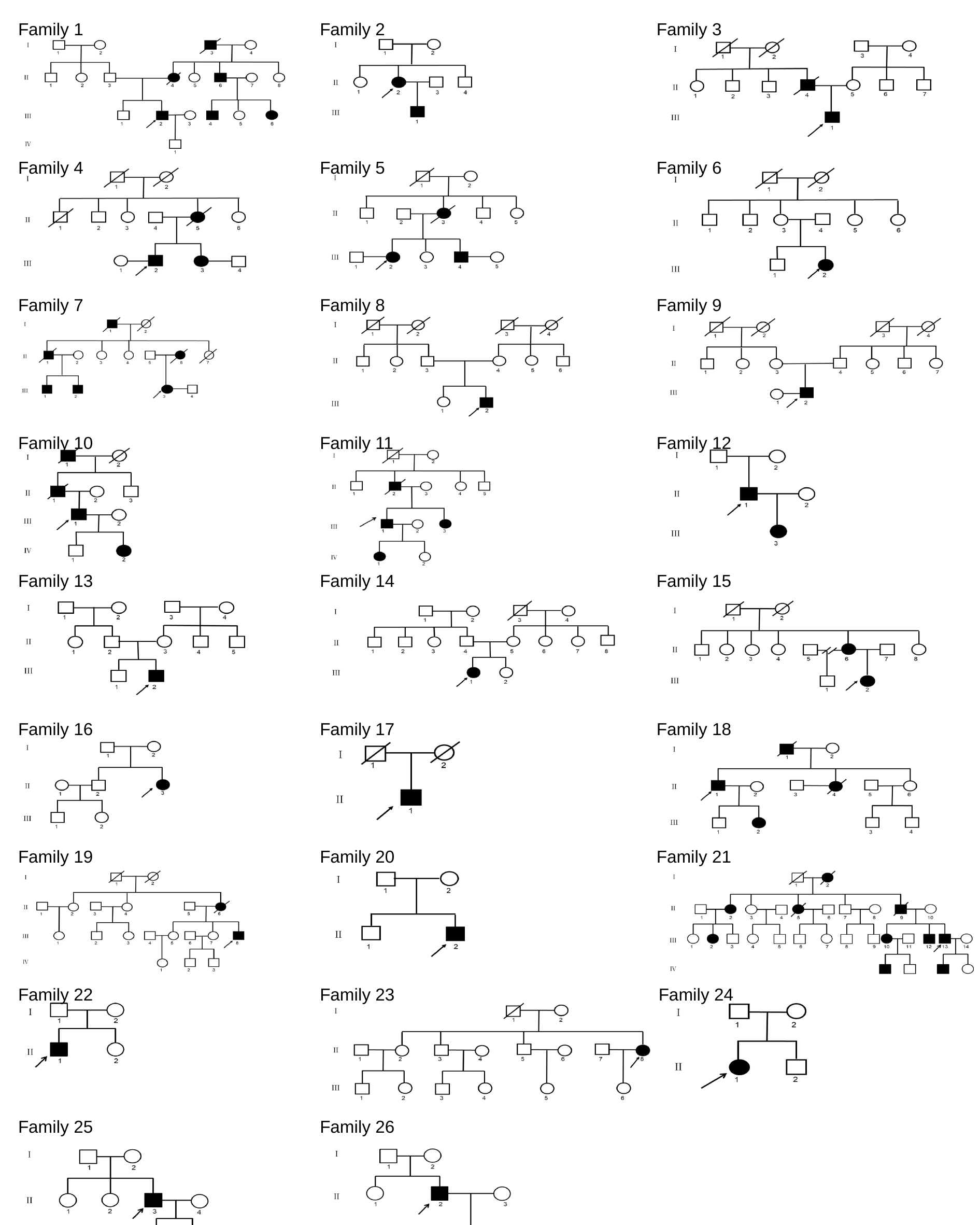

Family 1
Family 2
Family 3
Family 4
Family 5
Family 6
Family 7
Family 8
Family 9
Family 10
Family 11
Family 12
Family 13
Family 14
Family 15
Family 16
Family 17
Family 18
Family 19
Family 20
Family 21
Family 22
Family 23
Family 24
Family 25
Family 26
