# Supplementary material for: Clinical and Genetic Analyses of 38 Chinese Patients with Peutz-Jeghers Syndrome
Source: Biomed Res Int. 2020 May 11;2020:9159315. doi: 10.1155/2020/9159315 (PMC7240661; doi:10.1155/2020/9159315)
Supplement: Supplementary Materials — Supplementary Figure 1: The pedigree of 26 families. Supplementary Figure 2: Chromatogram of 11 other STK11 gene mutations. Supplementary Table 1: Primers of the STK11 gene. Supplementary Table 2: Detailed clinical and genetic data for patients with PJS. [file 9159315.f1.zip › mat.9159315.v2.pdf]

Supplementary Figures

Family 1

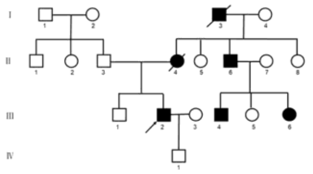

Family 2

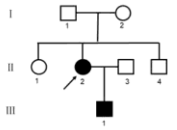

Family 3

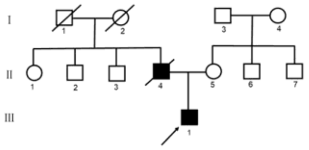

Family 4

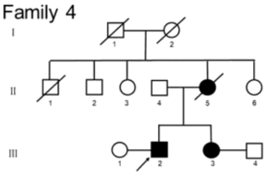

Family 5

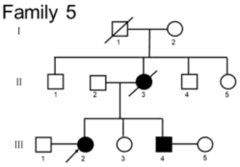

Family 6

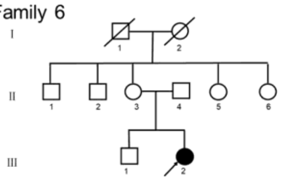

Family 7

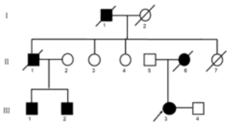

Family 8

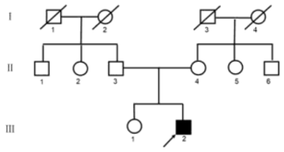

Family 9

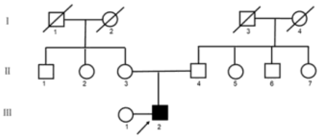

Family 10

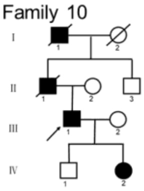

Family 11

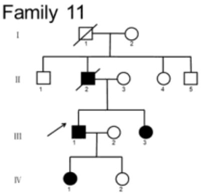

Family 12

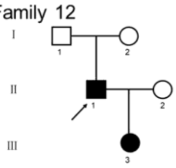

Family 13

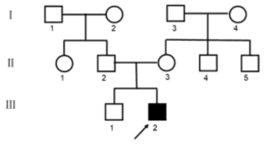

Family 14

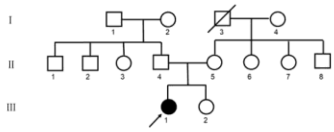

Family 15

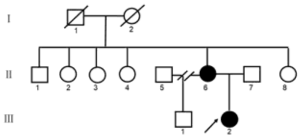

Family 16

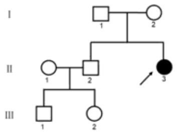

Family 17

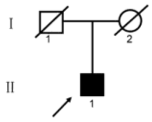

Family 18

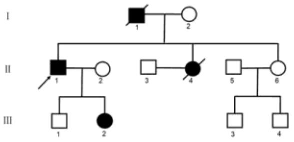

Family 19

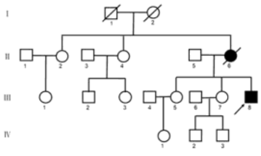

Family 20

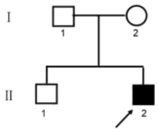

Family 21

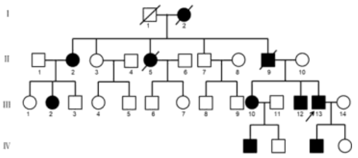

Family 22

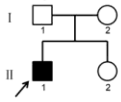

Family 23

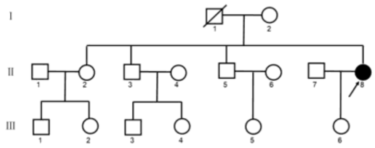

Family 24

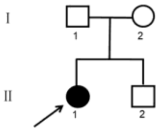

Family 25

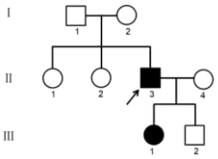

Family 26

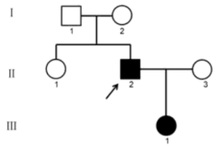

Supplementary Figure 1: The pedigree of 26 families. Family members are identified by generations and numbers. Circles and squares denote females and males, respectively; blackened symbols indicate the PJS patient; the proband is indicated by an arrow. Symbols with a slash indicate the individual was already dead.

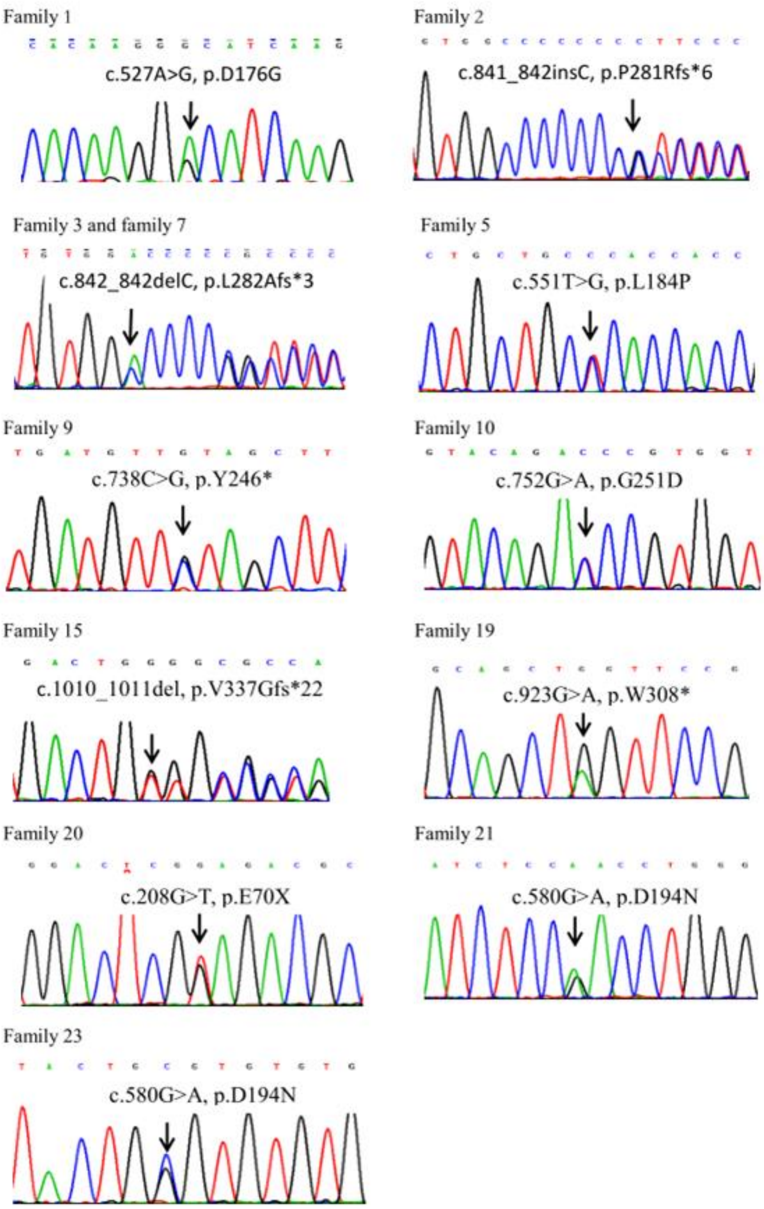

Supplementary Figure 2: Chromatogram of 11 other *STK11* gene mutations; mutations are indicated with arrows.

Table 1 Primers of *STK11* gene

	Forward primer	Reverse primer
Exon 1	5’-gggactgacgtgtagaacaatc-3’	5’-ggaaggaagacagaaccatcag-3’
Exon 2	5’-tctagggaagggaggaggta-3’	5’-tcaaggagacgggaagagg-3’
Exon 3	5’-cctgagctgtgtgtccttag-3’	5’-gggccttcatgtcaatgaatatc-3’
Exon 4-5	5’-cctttcctctgtcctgtgtg-3’	5’-ccatctgccgtatgagttacat-3’
Exon 6	5’-actgaccacgcctttcttc-3’	5’-tctccactcagtcctctcaat-3’
Exon 7	5’-ttaggagcgtccaggtatca-3’	5’-gccagtgtttgggaccag-3’
Exon 8	5’-catggctgagcttctgtgg-3’	5’-gaggctttggggacgtgggattgg-3’
Exon 9	5’-gggggcagcatttcagg-3’	5’-ggcatccaggcgttgtcc-3’

Supplementary table 2 Detailed clinical and genetic data for patients with PJS

Family	Mutation			Patient			Initial evaluation		First detection of polyps		Clinical course***			
ID	Exon	Nucleotide	Effect	ID	Sex*	Current Age(y	Age(y)	Main Reason*	Age(y)	Main Reason**	AP	GB	IB	Surgery
1	4	c.527A>G	p.D176G	1	M	26	20	MP	20	MP	Y	Y	Y	Y
				2	M	57	53	FH	–	–	N	N	N	N
				3	M	27	23	IB	15	IB	Y	Y	Y	Y
				4	F	9	5	FH	–	–	N	N	N	N
				5	M	2	0	FH	–	–	N	N	N	N
2	6	c.842_842insC	p.L282Afs*3	6	F	42	16	GB	16	GB	Y	Y	Y	Y
				7	M	18	14	GB	14	GB	Y	Y	N	N
3	6	c.841_842delC	p.P281Rfs*6	8	M	24	17	GB	17	GB	Y	Y	N	Y
4	7	c.889A>G	p.R297G	9	M	34	22	GB	22	GB	N	Y	N	Y
				10	F	31	25	FH	25	FH	Y	Y	N	N
5	4	c.551T>C	p.L184P	11	F	40	21	FH	21	FH	N	N	N	N
				12	M	35	20	FH	20	FH	N	N	N	N
6		Het del exon 1		13	F	32	23	IB	23	IB	Y	N	Y	Y
7	6	c.841_842insC	p.L282Afs*3	14	F	31	16	IB	16	IB	Y	Y	Y	Y
8		Het del exon 1		15	M	18	11	IB	11	IB	Y	Y	Y	Y
9	6	c.738C>G	p.Y246*	16	M	40	15	IB	15	IB	Y	N	Y	Y
10	6	c.752G>A	p.G251D	17	M	37	29	GB	29	GB	N	Y	N	N
11		Het del exon 1		18	M	35	26	IB	26	IB	Y	N	Y	Y
				19	F	33	20	FH	20	FH	Y	N	N	N
12	3	c.457_458insCC	p.H154Pfs*8	20	M	45	20	IB	20	IB	Y	N	Y	Y
				21	F	21	13	IB	13	IB	Y	N	Y	Y
13		Het del exon 2-3		22	M	9	2	GB	5	GB	Y	Y	N	N
14	3	c.393C>A	p.Y131*	23	F	22	15	IB	15	IB	Y	Y	Y	Y
15	8	c.1010_1011delTG	p.V337Gfs*22	24	F	21	17	GB	17	GB	N	Y	N	N
16		Het del exon 8		25	F	8	5	SV	5	SV	Y	N	N	Y
17	3	c.428_428delT	p.V143Gfs*18	26	M	19	2	GB	2	GB	Y	Y	Y	Y
18		Het del exon 2-3		27	M	50	43	GB	43	GB	Y	Y	N	N
19	8	c.923G>A	p.W308*	28	M	31	22	GB	22	GB	Y	Y	N	N
20	1	c.208G>T	p.E70*	29	M	18	11	IB	11	IB	Y	N	Y	Y
21	4	c.580G>A	p.D194N	30	M	32	14	IB	14	IB	Y	N	Y	Y
				31	M	28	27	FH	27	FH	N	N	N	N
22	5	c.601_601delC	L201Cfs*86	32	M	23	22	IB	22	IB	Y	N	Y	Y
23	3	c.396C>G	C132W	33	F	50	20	IB	20	IB	Y	Y	Y	Y
24	8	c.930delG	p. K311Rfs*25	34	F	7	6	MP	–	–	N	N	N	N
25	5	c.716G>C	p.W239S	35	M	34	20	D	20	D	Y	N	N	N
				36	F	9	9	FH	–	–	N	N	N	N
26	2	c.358G>T	p. E120*	37	M	31	10	MP	18	MP	Y	Y	Y	N
				38	F	4	4	FH	–	–	N	N	N	N

*M=male; F=female; **MP=mucocutaneous hyperpigmentation; FH=family history; GB=gastrointestinal bleeding; IB=intestinal obstruction; SV=Stomach Volvulus; D=diarrhea; "-"represents did not do any intestinal-associated testing; ***AP=abdominal pain; Y=yes; N=no
